# Reviewer acknowledgment 2015

**DOI:** 10.1186/s13019-016-0429-8

**Published:** 2016-02-22

**Authors:** Vipin Zamvar, David Taggart

**Affiliations:** Royal Infirmary of Edinburgh, Edinburgh, UK; John Radcliffe Hospital, Oxford, UK

## Abstract

The Editors of the *Journal of Cardiothoracic Surgery* would like to thank all of our reviewers who have contributed to the journal in Volume 10 (2015) and whose valuable support is fundamental to its success.

**Ahmad Abugameh**

Germany

**Anders Ahlsson**

Sweden

**Aslı Gül Akgül**

Turkey

**Rui Almeida**

Brazil

**Arangannal Arul**

UK

**Conal Austin**

UK

**Tara Baetz**

Canada

**Clifford Barlow**

UK

**Volkan Baysungur**

Turkey

**Pascal Berna**

France

**Jose M Bernal**

Spain

**Alessandro Bertani**

Italy

**Timothy Billiar**

USA

**Nikolaos Bonaros**

Austria

**Allison Cabalka**

USA

**Huseyin Altug Cakmak**

Turkey

**Antonio Calafiore**

Saudi Arabia

**Leonardo Canale**

Brazil

**Huiqing Cao**

China

**Burçin Çelik**

Turkey

**Liangwan Chen**

China

**Zhenguang Chen**

China

**Paola Ciriaco**

Italy

**Francisco Costa**

Brazil

**Abhishek Deshmukh**

USA

**Alpha Diallo**

UK

**Doa El-Ansary**

Australia

**Bachar El Oumeiri**

Belgium

**Wentao Fang**

China

**Yue Feng**

China

**Christophoros Foroulis**

Greece

**Changqing Gao**

China

**Roman Gottardi**

Austria

**Jibo Hu**

China

**Shengshou Hu**

China

**Antonio Lio**

Italy

**Changyu Liu**

Chile

**Alan Kirk**

UK

**Valerie Lacroix**

Belgium

**Yongqiang Lai**

China

**Harry Lapierre**

Canada

**Zhidong Liu**

China

**Haiyan Lou**

China

**Donna Maziak**

Canada

**John Moore**

Australia

**Henrique Murad**

Brazil

**Girish Nair**

Canada

**Vinicius Nina**

Brazil

**Robert Nolan**

Canada

**Maral Ouzounian**

Canada

**Bharat Pancholy**

USA

**Jason Peart**

Australia

**Mate Petricevic**

Croatia

**Claudio Picariello**

Italy

**Edvin Prifti**

Albania

**Eduardo Rocha**

Brazil

**Alfredo Rodrigues**

Brazil

**Alistair Royse**

Australia

**Fernando Sales**

Brazil

**Cleusa Santos**

Brazil

**Thomas Schachner**

Austria

**Pedro Silva**

Brazil

**James Tatoulis**

Australia

**Euclides Tenorio**

Brazil

**Pierre Voisine**

Canada

**Song Wan**

China

**Bing Yen Wang**

Taiwan

**Qing Wang**

China

**Xi Ming Wang**

China

**Atsushi Watanabe**

Japan

**Guangzhao Yang**

China

**Mohammed Yasin**

UK

**Can Yerebakan**

Germany

**Hong Yu**

China

